# Ecotoxicological Evaluation of Bisphenol A and Alternatives: A Comprehensive In Silico Modelling Approach

**DOI:** 10.3390/jox13040046

**Published:** 2023-11-23

**Authors:** Liadys Mora Lagares, Marjan Vračko

**Affiliations:** Laboratory for Cheminformatics, Theory Department, National Institute of Chemistry, 1000 Ljubljana, Slovenia; marjan.vracko@ki.si

**Keywords:** bisphenol A (BPA), BPA alternatives, ecotoxicity assessment, in silico models, principal component analysis (PCA), environmental impact

## Abstract

Bisphenol A (BPA), a compound widely used in industrial applications, has raised concerns due to its environmental impact. As a key component in the manufacture of polycarbonate plastics and epoxy resins used in many consumer products, concerns about potential harm to human health and the environment are unavoidable. This study seeks to address these concerns by evaluating a range of potential BPA alternatives, focusing on their ecotoxicological properties. The research examines 76 bisphenols, including BPA derivatives, using a variety of in silico ecotoxicological models, although it should be noted that these models were not developed exclusively for this particular class of compounds. Consequently, interpretations should be made with caution. The results of this study highlight specific compounds of potential environmental concern and underscore the need to develop more specific models for BPA alternatives that will allow for more accurate and reliable assessment.

## 1. Introduction

Bisphenol A (BPA) has long been a focal point of environmental concern due to its widespread use in various industrial applications, particularly in the production of plastics, resins, and thermal paper. It is valued for its ability to enhance the strength and flexibility of synthetic products. BPA is a key ingredient in producing polycarbonate plastics, epoxy resins, certain flame retardants, and various other materials [1,2]. BPA is found in things like plastic containers, metal can linings, packaging for cosmetics and personal care items, cookware, toys, receipts, sports gear, and medical devices [1,3]. It has even been detected in canned foods, drinks, and cosmetic and personal care products [4,5,6]. Its omnipresence in everyday products has raised significant concerns about potential harm to human health and the environment. Studies have linked BPA exposure to a range of health issues, including endocrine disruption, reproductive abnormalities, developmental disorders, and metabolic changes, among others [7,8,9,10,11]. Consequently, there is a growing need to explore alternative compounds that could be safer replacements for BPA.

**Figure 1:**
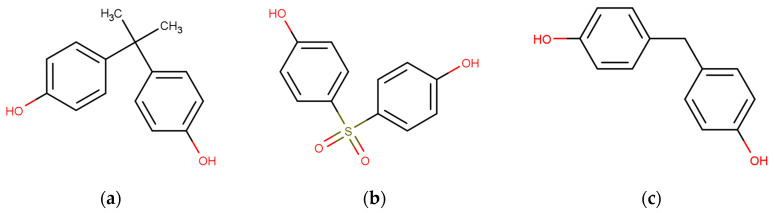
Structure of bisphenol A and some derivatives: (**a**) Bisphenol A; (**b**) Bisphenol S; (**c**) Bisphenol F.

In this context, a new generation of compounds meant to replace BPA has been developed. Some alternatives, including bisphenol F (BPF), bisphenol S (BPS), and bisphenol AF (BPAF), have been created and are now used in everyday products [8,12]. However, the new “BPA-free” alternatives closely resemble the original BPA in structure, with minor changes in certain parts of the molecule (Figure 1). While the term “BPA-free” may give the idea of safer options, it is important to note that the safety of these substitutes is not fully verified [12,13,14]. Due to their structural similarities with BPA, the alternatives may also have similar endocrine-disrupting effects, and many studies are reporting potential health risks associated with them [15,16].

Consequently, concerns about the environmental impact of BPA alternatives are growing. Compounds like BPS and BPF, which are replacing BPA in various products, are increasingly being detected in the environment [12], yet information about their impacts on ecosystems is scattered and incomplete. Given the escalating adoption of these BPA-free alternatives, it is crucial to assess their potential environmental impact. Understanding the ecotoxicity profile of BPA alternatives is essential in order to identify any potential risks to overall environmental health.

The investigation of BPA and its alternatives is intrinsically linked to regulatory and policy considerations. Governmental agencies and international organizations face the challenge of formulating evidence-based regulations and guidelines for the use of chemicals like BPA. Strong scientific research is essential in guiding these regulatory decisions, ensuring they safeguard public health and are based on solid ecological principles. This is why recent focus in the scientific community has shifted towards BPA-alternative research. For instance, the PARC project (partnership for the assessment of risks from chemicals) has also recognized the significance of this issue [17], incorporating it as a priority within its agenda.

Regulatory measures have already been implemented concerning BPA, which is acknowledged for its adverse health effects, leading to restrictions on its use. For example, BPA has been banned in various consumer products for infants and children since 2008 [18]. European Union countries have banned BPA in thermal paper when its concentration exceeds 0.02% [19]. Moreover, the European Food Safety Agency (EFSA), after reassessing the risks associated with BPA in food, recommended a reduction in its tolerable daily intake in 2021 [20]. Despite these actions, the potential toxicity of the alternatives to BPA has not been comprehensively evaluated due to the lack of experimental data and the observed variability in the experimental procedures when experimental data are available. In such cases, in silico toxicology methods emerge as valuable tools for addressing and bridging this gap. Currently, in silico approaches are frequently employed alongside other toxicity tests. Nevertheless, there is a growing trend in using these approaches to generate toxicity assessment information, reducing the necessity for in vitro or in vivo studies, depending on the decision context.

Over the past few years, in silico tools have become a key component in the drug development pipeline [21,22]. They have also gained significance as valuable tools for assessing ecotoxicity endpoints, offering a cost-effective, time-efficient, and ethically responsible alternative to conventional experimental approaches. They play a crucial role in predicting and evaluating the potential environmental impact of chemicals, thereby reducing the necessity for extensive animal testing. In fact, the use of non-animal alternative methods, especially in silico approaches, is playing a growing role in regulatory submissions. The European Union’s REACH regulation specifies acceptable alternative methods for data gap filling in Annex XI [23]. Similarly, the U.S. revised the Toxic Substances Control Act (TSCA) in 2016 to include predictive models and expert reviews as integral components of the overall assessment [24].

The FDA places emphasis on employing computational toxicology methods when experimental data are lacking in the evaluation of medical devices and electronic nicotine delivery devices [25,26]. Additionally, for chemicals with limited toxicity data, in silico toxicology offers a quick alternative for addressing data gaps in toxicity/safety information, aiding in classification and labeling for shipping and other purposes [27,28].

Within regulatory frameworks, a collaborative approach is evident, where in silico predictions complement standard toxicological data. This synergy is demonstrated by in silico data providing supporting information for primary in vivo or in vitro studies, offering a mechanistic understanding of the results, and contributing to a more precise definition of the experimental needs. In effect, in silico methods can guide or prioritize in vitro testing [29].

In silico tools employ algorithms and data analysis techniques to predict the potential environmental impact of chemicals on various organisms and ecosystems. By employing large datasets and advanced mathematical models, in silico methods can rapidly screen a plethora of compounds, prioritizing those with higher ecological risks for further analysis. Furthermore, they facilitate a deeper understanding of the underlying mechanisms governing toxicity, enable the design of safer chemicals, and aid in regulatory decision-making. Indeed, the European Commission’s Scientific Committee for Consumer Safety (SCCS), responsible for assessing the risk of cosmetic ingredients, recommends integrating in silico methods for internal decision-making or as part of a weight-of-evidence approach [30]. In silico tools represent a pillar in modern environmental risk assessment, contributing significantly to the sustainable conservation and protection of the environment.

However, existing in silico models are developed using compounds from diverse chemical categories and may encompass only individual bisphenols such as BPA, BPAF, and BPS in their training data. These models often also have a restricted set of ecotoxicological endpoints, which might not consistently meet the demands of risk assessment. At present, a multitude of QSAR models are at our disposal for predicting toxicity across a broad spectrum of compounds. However, there is a notable absence of specialized models tailored for the specific prediction of the ecotoxicological characteristics of bisphenols and their derivatives.

Within this framework, this study aims to comprehensively assess the ecotoxicological properties of BPA and its potential alternatives, providing a detailed insight into their potential impacts on the environment and human health. Our research outlines the in silico tools employed to evaluate the bioactivity of BPA alternatives, focusing on the use of existing models for different relevant environmental endpoints. This approach allows us to gain a comprehensive understanding of the available tools and their limitations, contributing to the identification of needs for the development of new methods—an area of crucial focus for the PARC project. Investigating BPA and exploring viable alternatives is of social and environmental significance. This exploration goes beyond immediate health concerns and looks into broader goals related to sustainability, human well-being, and ecological integrity.

## 2. Materials and Methods

### 2.1. Dataset

The data set consists of 76 bisphenols, which are considered potential alternatives for bisphenol A (see Supplementary Materials in Table S1). Among them, four compounds have disconnected structures (ionized form). Their structures, encoded in SMILES notations, have been used to calculate 708 descriptors using the alvaDesc version 2.0.10 tool, which calculates 33 different groups of descriptors [31,32]. One of the most innovative features of alvaDesc is its capability to handle both full-connected and non-full-connected molecular structures, such as salts and ionic liquids. All the molecular descriptor calculation algorithms provide different theoretical approaches for the calculation of molecular descriptors on such structures. During this calculation, we considered descriptors derived from zero, one, and two-dimensional structures. Any descriptors that were unavailable for certain molecules were intentionally excluded from the analysis. Additionally, the descriptors were also calculated for the four compounds characterized by disconnected structures. Subsequently, the number of descriptors was reduced with the principal component analysis (PCA) method.

### 2.2. Principal Component Analysis

Principal component analysis (PCA) is a widely employed technique for analyzing multi-dimensional data. The basic idea of PCA is to reduce the number of variables while retaining the essential information within the dataset. Mathematically, PCA involves a linear transformation of the original variables into new, latent variables. The transformation matrix is derived from the eigenvectors of the variance matrix, which are organized based on their information content. This means that objects are effectively represented in a new space with only a few variables.

The outcomes of PCA are typically presented through three key components. The informational content of individual latent variables, as well as the cumulative informational content, is commonly presented in a scree plot. The objects presented in the latent variables are visualized in score plots. In our specific case, these representations are color-coded in accordance with their predicted properties. Furthermore, the loadings analysis is employed to assess which original variables exert significant influence on a given latent variable.

### 2.3. Ecotox Models Implemented in Vega and Their Suitability for Predicting BPA Alternatives

We assessed the eco-toxicological properties of BPA and derivatives using the Ecotox models integrated within the online platform VEGAHUB 2013 [33], which are freely available to the public at https://www.vegahub.eu/portfolio-item/vega-qsar/ (accessed on 3 October 2023).

Specifically, we focused on models that included one or more bisphenol derivatives in their training data. The relevant models and associated bisphenols can be found in Table 1. It is important to note that the four compounds with disconnected structures were not predicted by the models.

#### 2.3.1. Fish Acute (LC50) Toxicity Model (IRFMN) Version 1.0.1 [34,35]

This model provides a quantitative prediction for toxicity in fish (*Oryzias latipes*, Japanese ricefish/medaka) LC50 (96 h), measured in mg/L. The model is constructed using a tree ensemble random forest approach and is based on data from the Japanese Ministry of Environment dataset [36].

The model’s endpoint is focused on short-term toxicity to fish, specifically utilizing the OECD Test No. 203 fish acute toxicity test, which evaluates the mortalities after exposure to the test substance, preferably for a period of 96 h.

The dataset used to build the model comes from the Japanese Ministry of Environment and comprises 331 experimental data points on *Oryzias latipes* selected according to OECD Test No. 203. To create the QSAR model, the dataset was divided into training and test sets in an 80:20 ratio. In this model, the training set includes two bisphenols: BPA alternatives, such as BPA and BPF. This inclusion enhances the model’s capacity to predict this endpoint in compounds with similar structural characteristics, but it is not enough to ensure robust and reliable predictions for this specific class of compounds.

#### 2.3.2. Fathead Minnow LC50 (96 h) (EPA) Version 1.0.7 [37,38]

The model provides a quantitative prediction for fathead minnow (*Pimephales promelas*) LC50 (96 h) in mg/L. It is a re-implementation of Todd Martin’s original model developed within the T.E.S.T. version 5.1.2 software for the US EPA.

The model’s endpoint is focused on short-term toxicity to fish. The fathead minnow LC50 endpoint represents the concentration in water, which kills half of fathead minnow (*Pimephales promelas*) in 4 days (96 h).

The model is a linear regression model based on 21 molecular descriptors. The regression coefficients were calculated using the original T.E.S.T. dataset, which consists of 816 compounds extracted from the ECOTOX aquatic toxicity database (http://cfpub.epa.gov/ecotox/) (accessed on 3 October 2023). While the training set does include BPA and TBBPA, it is essential to pay special attention when analyzing predictions made on this class of compounds. The limited presence of these compounds in the training set may not be sufficient to ensure robust and reliable predictions.

#### 2.3.3. Daphnia Acute (EC50) Toxicity Model (IRFMN) Version 1.0.1 [39]

The model provides a quantitative prediction of acute toxicity in *Daphnia Magna* (EC50), given in mg/L. The model is constructed using a tree ensemble random forest approach and is based on data from the Japanese Ministry of Environment dataset [36].

The endpoint used for the models was the short-term toxicity to aquatic invertebrates, specifically the 48 h *Daphnia magna* EC50 according to OECD Test No. 202: Daphnia sp. acute immobilization test. This test assesses the percentage of immobilized daphnids after a 48 h exposure to a test compound.

The dataset comprises 445 experimental data retrieved from the Japanese Ministry of Environment, selected according to OECD Test No. 202, and was split randomly into the training and test sets. In this model, the training set encompasses different BPA alternatives, such as BPA, BPS, BPF, BPZ, and TBBPA. This inclusion enhances the model’s capacity to predict this endpoint in compounds with similar structural characteristics.

#### 2.3.4. Algae Acute EC50 Toxicity Model (IRFMN) Version 1.0.1 [35,40]

This model provides a quantitative prediction for toxicity in algae (*Raphidocelis subcapitata*) EC50 (72 h), measured in mg/L. The model is constructed using a Tree Ensemble Random Forest approach and is based on data from the Japanese Ministry of Environment dataset [36].

The model’s endpoint is focused on long-term toxicity to aquatic algae and cyanobacteria, specifically utilizing the OECD Test No. 201, freshwater alga and cyanobacteria, growth inhibition test. This test evaluates the growth inhibition of algae and cyanobacteria when exposed to different compounds.

The dataset used to build the model comes from the Japanese Ministry of Environment and comprises 315 experimental data points on algae acute toxicity ErC50 values. These values represent the reduction in growth rate (ErC50) and are expressed in mg/L after 72 h. In this model, the training set encompasses different BPA alternatives, such as BPA, BPA 2 EO, BPF, TBBPA, and 4,4′,4″-(ethan-1,1,1-triyl)triphenol. This inclusion enhances the model’s capacity to predict this endpoint in compounds with similar structural characteristics.

#### 2.3.5. Algae (EC50) Toxicity Model (ProtoQSAR/Combase) Version 1.0.1 [41,42]

This model provides a quantitative prediction for toxicity in algae (*Raphidocelis subcapitata*) EC50 (72 h), measured in mg/L. The model is constructed using a support vector machine approach and is based on data from the Japanese Ministry of Environment dataset [36], including experimental values of ErC50 after 72 h for *R. subcapitata* for 361 mono-constituent organic biocide-like compounds.

The model’s endpoint is focused on long-term toxicity to aquatic algae and cyanobacteria, specifically utilizing the OECD Test No. 201, freshwater alga and cyanobacteria, growth inhibition test. This test evaluates the growth inhibition of algae and cyanobacteria when exposed to different compounds.

The whole dataset was randomly divided into a training set (70%), a validation set (15%), and an external validation set (15%). The training set includes BPA, BPA 2 EO, BPS, BPF, BPZ, TBBPA, and 4,4′,4″-(ethan-1,1,1-triyl)triphenol, which enhances the model’s ability to predict this endpoint in compounds with similar structural characteristics.

#### 2.3.6. Algae Chronic (NOEC) Toxicity Model (IRFMN) Version 1.0.1 [43]

This model provides a quantitative prediction for toxicity in algae (*Raphidocelis subcapitata*) EC50 (72 h), measured in mg/L. The model is constructed using the tree ensemble random forest approach and is based on experimental values of algae chronic toxicity (NOEC, 72 h growth rate) for 410 compounds retrieved from the Japanese Ministry of Environment dataset [36].

The model’s endpoint is focused on long-term toxicity to aquatic algae and cyanobacteria, specifically utilizing the OECD Test No. 201, freshwater alga and cyanobacteria, growth inhibition test. This test evaluates the growth inhibition of algae and cyanobacteria when exposed to different compounds.

To derive the models, the dataset was divided into training and test sets with a ratio of 80:20. In this model, the training set encompasses different BPA alternatives, such as BPA, BPA 2EO, BPF, and BPZ. This inclusion enhances the model’s capacity to predict this endpoint in compounds with similar structural characteristics.

#### 2.3.7. Sludge Classification Toxicity Model for Biocides (ProtoQSAR/COMBASE) Version 1.0.0 [44,45]

The model provides a binary classification model designed specifically to predict toxicity in activated sludge, with a focus on biocides. The model was built using the boosted trees algorithm based on experimental results on 94 biocide-like compounds for EC50 after 3 h on activated sludge from the COMBASE dataset and various databases within the OECD QSAR Toolbox, v. 4.2. (www.qsartoolbox.org) (accessed on 3 October 2023). Its development took place within LIFE15 ENV/ES/416.

The model’s endpoint relied on data from OECD Test No. 209: Respiration Inhibition. This method assesses the impact of a substance on microorganisms found in activated sludge, primarily bacteria. It measures the respiration rate of these microorganisms, which involves the oxidation of carbon and/or ammonium under specific conditions with varying concentrations of the test substance. During the test, samples of activated sludge with the test substance, as well as blank controls without the substance, are incubated with synthetic sewage. After a contact time of 3 h, the respiration rates are measured in an enclosed cell containing an oxygen electrode.

Two classes were defined based on the EC50 (3 h) < 100 mg/L threshold for toxic substances. Even though the training set comprises BPS and 4,4′,4″-(ethan-1,1,1-triyl)triphenol, their limited presence might not be adequate to guarantee strong and dependable predictions for this particular class of compounds.

#### 2.3.8. Sludge (EC50) Toxicity Version (ProtoQSAR/COMBASE) 1.0.1 [45,46]

This model provides a quantitative prediction for toxicity in activated sludge at EC50 (3 h), measured in mg/L. The model was built using multiple linear regression based on experimental results on 94 biocide-like compounds for EC50 after 3 h on activated sludge from the COMBASE dataset and various databases within the OECD QSAR Toolbox, v. 4.2. (www.qsartoolbox.org) (accessed on 3 October 2023). Its development took place within LIFE15 ENV/ES/416.

The classification model’s endpoint relied on data from OECD Test No. 209: respiration inhibition. This method assesses the impact of a substance on microorganisms found in activated sludge, primarily bacteria. It measures the respiration rate of these microorganisms, which involves the oxidation of carbon and/or ammonium under specific conditions with varying concentrations of the test substance. During the test, samples of activated sludge with the test substance, as well as blank controls without the substance, are incubated with synthetic sewage. After a contact time of 3 h, the respiration rates are measured in an enclosed cell containing an oxygen electrode.

Even though the training set comprises a bisphenol derivative: 4,4′,4″-(ethan-1,1,1-triyl)triphenol, their limited presence might not be adequate to guarantee strong and dependable predictions for this particular class of compounds.

#### 2.3.9. Bioconcentration Factors (BCF) Model (CAESAR) Version 2.1.15 [47,48,49]

The model provides a quantitative prediction of bioconcentration factor (BCF) in fish (*Cyprinos Carpio* and salmonids), given in log(L/kg). The hybrid model is constructed using multiple linear regression and radial basis function neural network approaches and is based on experimental log BCF values of 511 from Dimitrov et al. [50]

The model’s endpoint is focused on the bioconcentration potential of substances in fish, specifically utilizing OECD Test No. 305 bioaccumulation in fish: aqueous and dietary exposure. Bioconcentration occurs when a chemical concentration in an aquatic organism surpasses that in the surrounding water due to direct exposure, excluding dietary intake. This is quantified using a bioconcentration factor (BCF), represented by the ratio of the chemical concentration in the organism (CB) to that in the water (CW) at a steady state, where CB remains constant over time.

The model extends the original CAESAR model, previously available at http://www.caesar-project.eu, no longer supported. In this model, the training set encompasses BPA and TBBPA. This inclusion enhances the model’s capacity to predict this endpoint in compounds with similar structural characteristics, but their limited presence might not be adequate to guarantee strong and reliable predictions for this class of compounds.

#### 2.3.10. Bioconcentration Factors (BCF) Model (Arnot-Gobas) Version 1.0.1 [51,52]

The model provides a quantitative prediction of bioconcentration factor (BCF) in fish (*Cyprinos Carpio* and salmonids), given in log(L/kg). The hybrid model is constructed using multiple linear regression and radial basis function neural network approaches and is based on experimental log BCF values of 511 from Dimitrov et al. [50]

The model’s endpoint is focused on the bioconcentration potential of substances in fish, specifically utilizing OECD Test No. 305 bioaccumulation in fish: aqueous and dietary exposure. Bioconcentration occurs when a chemical concentration in an aquatic organism surpasses that in the surrounding water due to direct exposure, excluding dietary intake. This is quantified using a bioconcentration factor (BCF), represented by the ratio of the chemical concentration in the organism (CB) to that in the water (CW) at a steady state, where CB remains constant over time.

This model is an implementation of the Arnot-Gobas BAF-BCF model of EPISUITE. In this model, the training set includes BPA, TBBPA, and TBMD, enhancing predictions for structurally similar compounds. However, the limited data might not be enough to guarantee strong and reliable predictions for this class of compounds.

#### 2.3.11. Bioconcentration Factors (BCF) Model (Meylan) Version 1.0.4 [53,54]

The model provides a quantitative prediction of bioconcentration factor (BCF) in fish, given in log(L/kg). The model is based on the method proposed by Meylan et al. [54] as implemented in the EPI Suite BCFBAF module v 4.11. (http://www.epa.gov/oppt/exposure/pubs/episuite.htm) (accessed on 3 October 2023). The model provides a BCF prediction based on different regression equations or fixed values, selected on the basis of an initial classification between ionic and non-ionic compounds and on the value of the predicted logP value. The final dataset has 662 compounds.

The model’s endpoint is focused on the bioconcentration potential of substances in fish, specifically utilizing OECD Test No. 305 bioaccumulation in fish: aqueous and dietary exposure. The bioconcentration factor (BCF) is the concentration of the test substance in the fish or specified tissues thereof divided by the concentration of the chemical in the surrounding medium at steady state.

In this model, the training set includes BPA and TBBPA, enhancing predictions for structurally similar compounds. However, the limited data might not be enough to guarantee strong and reliable predictions for this class of compounds.

#### 2.3.12. Bioconcentration Factors (BCF) Model (kNN/Read-Across) Version 1.1.1 [55,56]

The model performs a read-across and provides a quantitative prediction of bioconcentration factor (BCF) in fish, given in log(L/kg). The read-across model has been built with the istKNN application and is based on the similarity index developed inside the VEGA platform; the index takes into account several structural aspects of the compounds.

The model’s endpoint is focused on the bioconcentration potential of substances in fish, specifically utilizing OECD Test No. 305 bioaccumulation in fish: aqueous and dietary exposure. The bioconcentration factor (BCF) is the concentration of the test substance in the fish or specified tissues thereof divided by the concentration of the chemical in the surrounding medium at steady state.

This dataset comprising 860 chemicals has been made by Istituto di Ricerche Farmacologiche Mario Negri, merging experimental data from several reliable sources, including the original dataset of the CAESAR BCF model. In this model, the training set includes BPA and TBBPA, enhancing predictions for structurally similar compounds. However, the limited data might not be enough to guarantee strong and reliable predictions for this class of compounds.

#### 2.3.13. Persistence (Soil) Quantitative Model (IRFMN) Version 1.0.1 [57]

The model is based on half-life ultimate biodegradation test data and provides an evaluation of persistence properties in the water compartment based on a dataset from Gouin et al. [58]

The model’s endpoint is focused on persistence in soil measured as half-life in days, specifically utilizing OECD Test No. 307: aerobic and anaerobic transformation in soil. This test determines the rate of transformation of the test substance and the nature and rates of formation and decline of transformation products to which plants and soil organisms may be exposed.

The training set of the model includes TBBPA, enhancing predictions for structurally similar compounds. However, the limited data might not be enough to guarantee strong and reliable predictions for this class of compounds.

**Table 1:** Ecotoxicological models available in VEGA version 1.1.5 software.

Model Name	Biological Model	Endpoint	Bisphenol Derivatives	Reference
Fish Acute (LC50) Toxicity model (IRFMN)	*Oryzias latipes* (*Japanese rice fish*)	Short-term toxicity to fish. Fish, Acute Toxicity Test	BPA ^1^, BPF ^2^	Toma et al., 2021 [35] https://www.vegahub.eu/vegahub-dwn/qmrf/QMRF_FISH_LC50_IRFMN.pdf (accessed on 5 October 2023)
Fathead Minnow LC50 96 h (EPA)	*Pimephales promelas* (*Fathead minnow*)	Short-term toxicity to fish	BPA, TBBPA ^3^	Martin et al., 2001 [38] https://www.vegahub.eu/vegahub-dwn/qmrf/QMRF_FATHEAD_LC50_EPA.pdf (accessed on 27 July 2023)
Daphnia Acute (EC50) toxicity model (IRFMN)	*Daphnia magna*	Short-term toxicity to aquatic invertebrates. Acute Immobilization Test	BPA, BPS ^4^, BPF, BPZ ^5^, TBBPA	https://www.vegahub.eu/vegahub-dwn/qmrf/QMRF_DAPHNIA_EC50_IRFMN.pdf (accessed on 5 October 2023)
Algae Acute (EC50) Toxicity model (IRFMN)	*Raphidocelis subcapitata* (*Pseudokirchneriella subcapitata*)	Long-term toxicity to aquatic algae and cyanobacteria C.f. OECD TG 201 Freshwater Alga and Cyanobacteria, Growth Inhibition Test	BPA, BPA 2 EO ^6^, BPF, TBBPA, 4,4′,4″-(ethan-1,1,1-triyl)triphenol	Toma et al., 2021 [35] https://www.vegahub.eu/vegahub-dwn/qmrf/QMRF_ALGAE_EC50_IRFMN.pdf (accessed on 27 July 2023)
Algae (EC50) Toxicity Model (ProtoQSAR/Combase)	*Raphidocelis subcapitata* (*Pseudokirchneriella subcapitata*)	Long-term toxicity to aquatic algae and cyanobacteria C.f. OECD TG 201 Freshwater Alga and Cyanobacteria, Growth Inhibition Test	BPA, BPA 2 EO, BPS, BPF, BPZ, TBBPA, 4,4′,4″-(ethan-1,1,1-triyl)triphenol	Blázquez et al. 2021 [41] https://www.vegahub.eu/vegahub-dwn/qmrf/QMRF_ALGAE_EC50_COMBASE.pdf (accessed on 5 October 2023)
Algae Chronic (NOEC) Toxicity model (IRFMN)	*Raphidocelis subcapitata* (*Pseudokirchneriella subcapitata*)	Long-term toxicity to aquatic algae and cyanobacteria C.f. OECD TG 201 Freshwater Alga and Cyanobacteria, Growth Inhibition Test	BPA, BPA 2EO, BPF, BPZ	https://www.vegahub.eu/vegahub-dwn/qmrf/QMRF_ALGAE_NOEC_IRFMN.pdf (accessed on 5 October 2023)
Sludge Classification Toxicity model (ProtoQSAR/COMBASE)	*Activated sludge*	Activated Sludge, Respiration Inhibition Test (OECD 209)	BPS, 4,4′,4″-(ethan-1,1,1-triyl)triphenol	https://www.vegahub.eu/vegahub-dwn/qmrf/QMRF_SLUDGE_CLASS_COMBASE.pdf (accessed on 27 July 2023)
Sludge (EC50) toxicity (ProtoQSAR/COMBASE)	*Activated sludge*	Activated Sludge, Respiration Inhibition Test (OECD 209)	4,4′,4″-(ethan-1,1,1-triyl)triphenol	https://www.vegahub.eu/vegahub-dwn/qmrf/QMRF_SLUDGE_EC50_COMBASE.pdf (accessed on 6 October 2023)
BCF model (CAESAR)	*Cyprinos Carpio* and *salmonids*	BCF fish	BPA, TBBPA	Zhao et al., 2008 [49] https://www.vegahub.eu/vegahub-dwn/qmrf/QMRF_BCF_CAESAR.pdf (accessed on 6 October 2023)
BCF model (Arnot-Gobas)	*Oncorhynchus mykiss* (*Rainbow trout*)	BCF fish	BPA, TBBPA, TBMD ^7^	Arnot et al., 2003 [51] https://www.vegahub.eu/vegahub-dwn/qmrf/QMRF_BCF_ARTNOTGOBAS.pdf (accessed on 6 October 2023)
BCF model (Meylan)	*Fish*	BCF fish	BPA, TBBPA	Meylan et al., 1999 [54] https://www.vegahub.eu/vegahub-dwn/qmrf/QMRF_BCF_MEYLAN.pdf (accessed on 6 Oc-tober 2023)
BCF model (kNN/Read-Across)	*Fish*	BCF fish	BPA, TBBPA	Manganaro et al., 2016 [56] https://www.vegahub.eu/vegahub-dwn/qmrf/QMRF_BCF_KNN.pdf (accessed on 6 October 2023)
Persistence (soil) quantitative model (IRFMN)	Soil	Biodegradation in soil. Aerobic and Anaerobic Transformation in Soil	TBBPA	https://www.vegahub.eu/vegahub-dwn/qmrf/QMRF_PERSISTENCE_SOIL_REG.pdf (accessed on 6 October 2023)

^1^ Bisphenol A. ^2^ Bisphenol F. ^3^ Tetrabromobisphenol A. ^4^ Bisphenol S. ^5^ Bisphenol Z. ^6^ Bisphenol A bis(2-hydroxyethyl)ether. ^7^ 4,4′-Methylenebis(2,6-DI-tert-butylphenol).

## 3. Results and Discussion

In the initial phase of our methodology, we employed chemometric tools to analyze a set of structurally diverse compounds. A total of 708 structural descriptors, devoid of ecotoxicological information, were utilized to encode these compounds. Subsequently, principal component analysis (PCA) was applied to compress the data and generate visual representations. Moving to the second phase, we aimed to predict various ecotoxicological properties using thirteen available in silico models.

To include the predictive results into PCA score plots, a color scheme was adopted, serving as an indicator of the level of concern associated with each compound. Specifically, we employed four models for predicting bioconcentration factors (BCF), three for evaluating algae toxicity, two for assessing fish toxicity, two for evaluating activated sludge toxicity, one for evaluating toxicity in *Daphnia magna*, and one for assessing toxicity in soil. This two-step approach not only facilitated a comprehensive analysis of the structural features of the compounds but also enabled the construction of an ecotoxicological profile for BPA alternatives based on the predictions obtained. This comprehensive strategy enhances our understanding of the potential environmental impact of the studied compounds.

**Figure 2:**
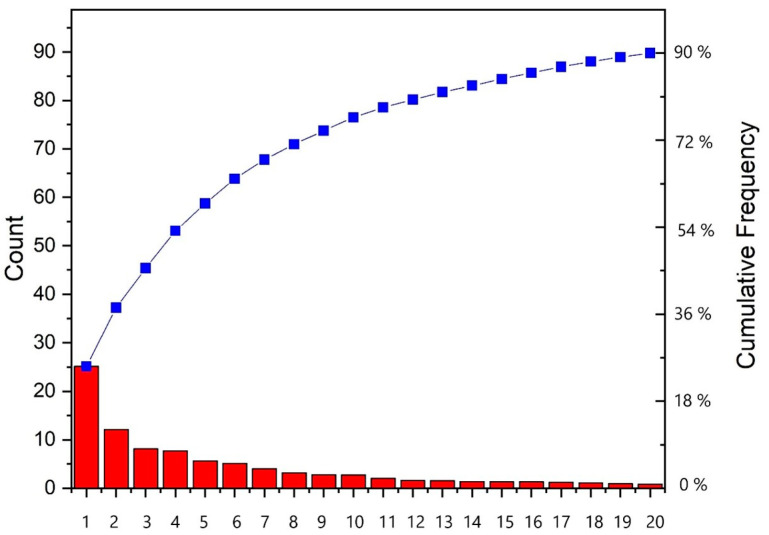
Scree plot showing the individual contributions to the total variance for first 20 PCs (red columns) and the cumulative variance (blue line).

In the scree plot of PCA shown in Figure 2, the first and second principal components (PCs) carry 25.14 and 12.08% of the total variance, respectively, while the first 20 PCs cumulatively carry 89.74% of the total variance.

Notably, there are no loadings with particularly higher values. Out of the 708 descriptors, 151 and 200 of them exhibit loading values higher than the average for PC1 and PC2, respectively. This implies that no particular descriptor group carries the essential part of the variance within the dataset.

**Figure 3:**
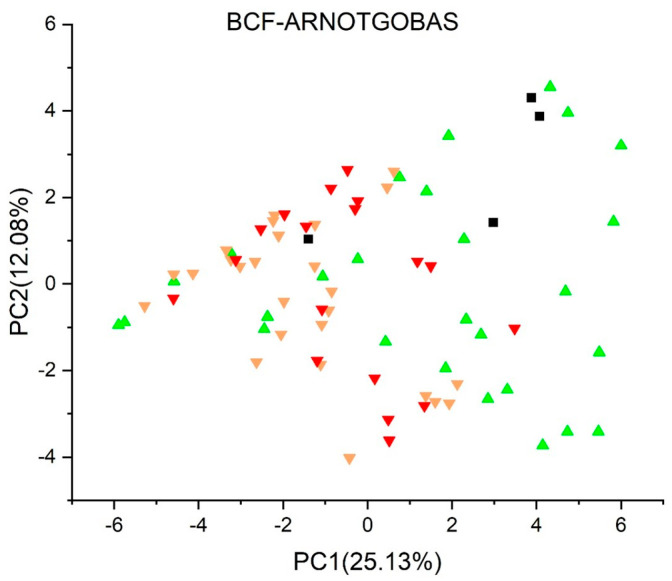
Score plot for BCF model (Arnot−Gobas). Compounds are color−coded based on their BCF values: green for values below 100 L/kg (considered of no concern), yellow for values between 100 and 1000 L/kg, and red for values exceeding 1000 L/kg. Compounds with disconnected structures are indicated by black squares.

**Figure 4:**
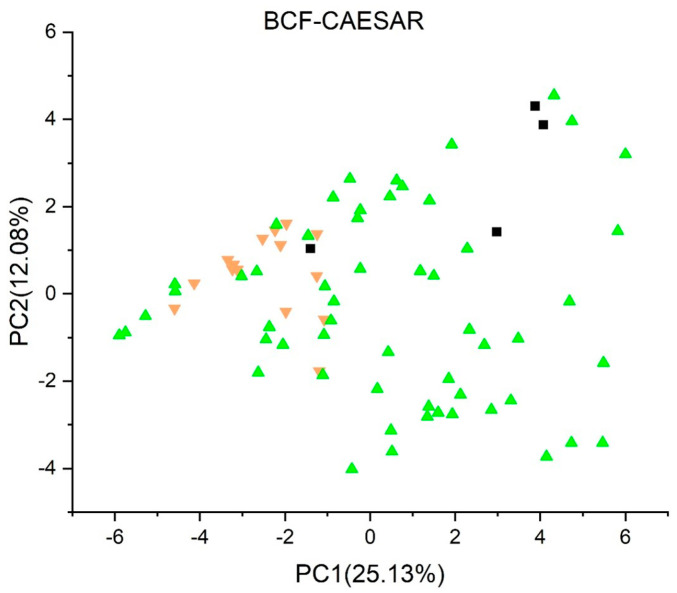
Score plot for BCF model (CAESAR). Compounds are color−coded based on their BCF values: green for values below 100 L/kg (considered of no concern), yellow for values between 100 and 1000 L/kg, and red for values exceeding 1000 L/kg. Compounds with disconnected structures are indicated by black squares.

**Figure 5:**
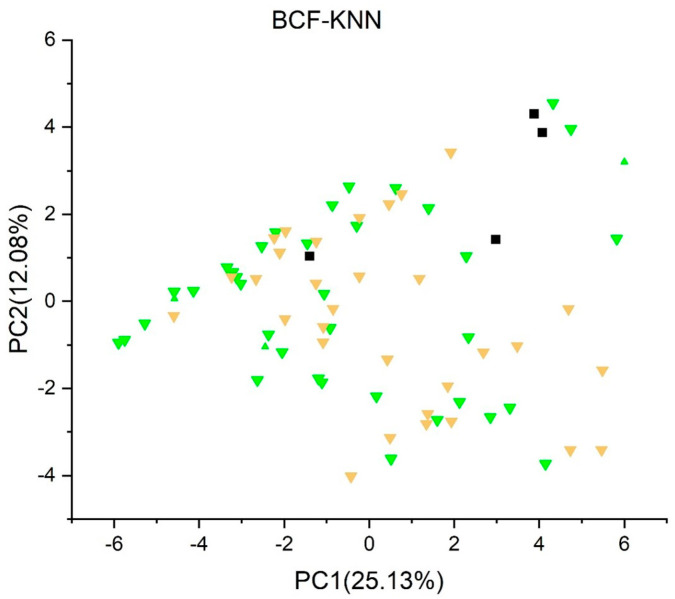
Score plot for BCF model (kNN/Read Across). Compounds are color−coded based on their BCF values: green for values below 100 L/kg (considered of no concern), yellow for values between 100 and 1000 L/kg, and red for values exceeding 1000 L/kg. Compounds with disconnected structures are indicated by black squares.

**Figure 6:**
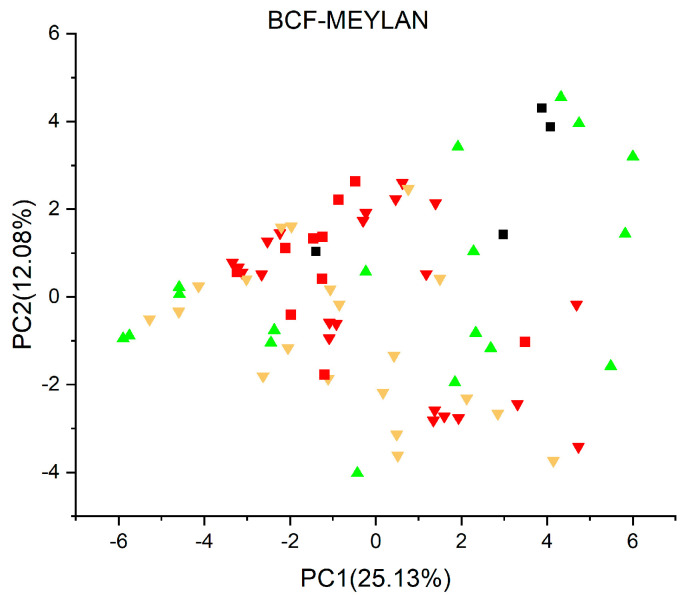
Score plot for BCF model (Meylan). Compounds are color−coded based on their BCF values: green for values below 100 L/kg (considered of no concern), yellow for values between 100 and 1000 L/kg, and red for values exceeding 1000 L/kg. Compounds with BCF larger than 10,000 L/kg are labeled with red squares. Compounds with disconnected structures are indicated by black squares.

**Figure 7:**
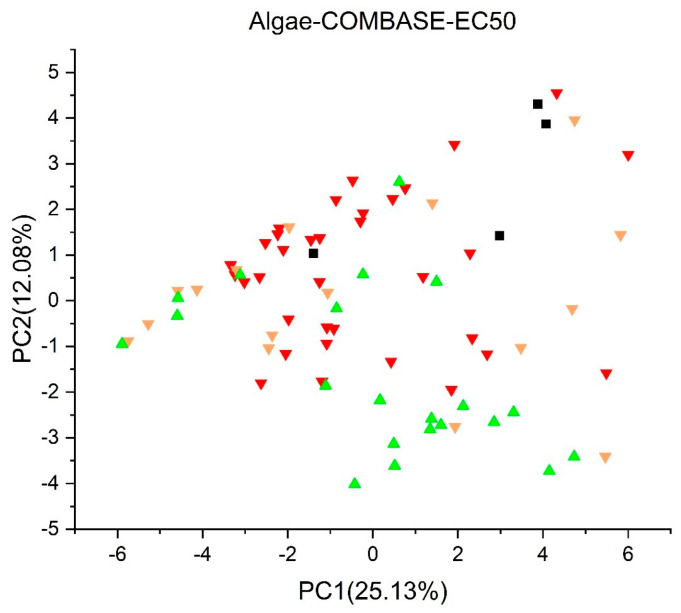
Score plot for Algae (EC50) Toxicity Model (ProtoQSAR/Combase). Compounds are color−coded based on their EC50 values: less than 1.0 mg/L are denoted in red, yellow for values between 1.0 and 10 mg/L, and green for values greater than 10 mg/L. Compounds with disconnected structures are indicated by black squares.

**Figure 8:**
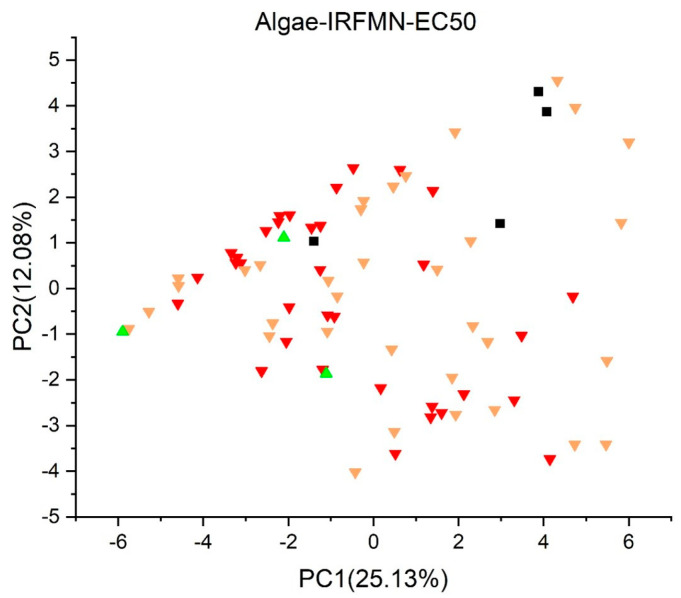
Score plot for Algae (EC50) Toxicity Model (IRFMN). Compounds are color−coded based on their EC50 values: less than 1.0 mg/L are denoted in red, yellow for values between 1.0 and 10 mg/L, and green for values greater than 10 mg/L. Compounds with disconnected structures are indicated by black squares.

**Figure 9:**
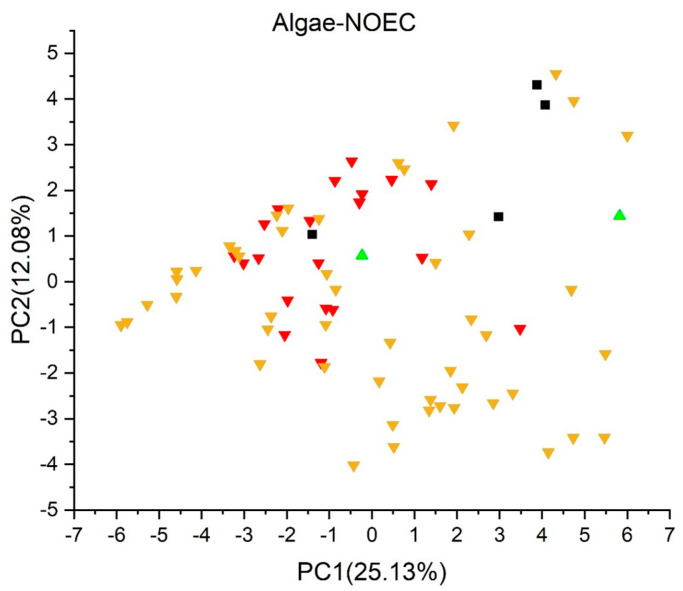
Score plot for Algae Chronic (NOEC) Toxicity model (IRFMN). Compounds are color−coded based on their NOEC values: under 0.1 mg/L in red, between 0.1 mg/L and 1.0 mg/L in yellow, and over 1.0 mg/L in green. Compounds with disconnected structures are indicated by black squares.

**Figure 10:**
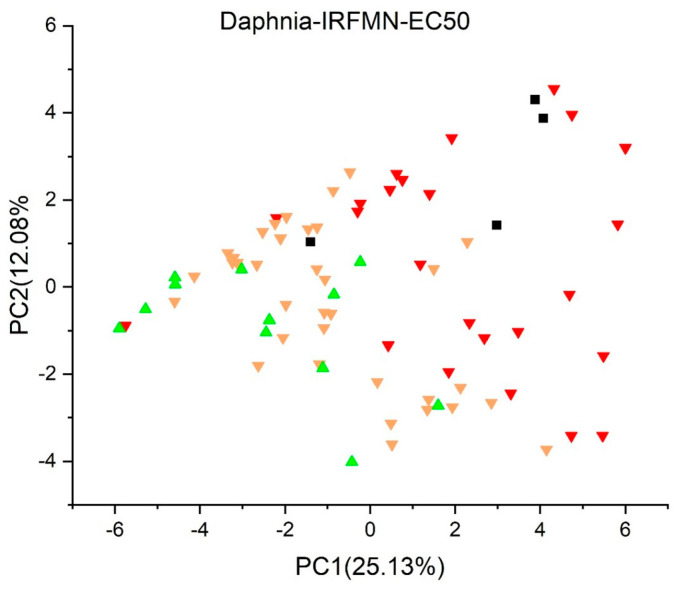
Score plot for Daphnia Acute (EC50) toxicity model (IRFMN). Compounds are color-coded based on their EC50 values: under 0.1 mg/L in red, between 0.1 mg/L and 1.0 mg/L in yellow, and over 1.0 mg/L in green. Compounds with disconnected structures are indicated by black squares.

**Figure 11:**
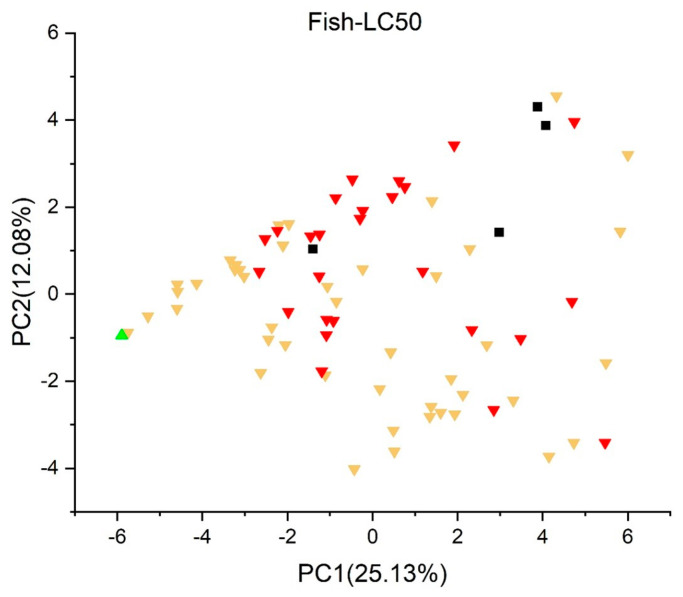
Score plot for fish acute (LC50) toxicity model (IRFMN). Compounds are color-coded based on their LC50 values: toxicity below 1 mg/L is in red, between 1 mg/L and 10 mg/L in yellow, and over 10 mg/L in green. Compounds with disconnected structures are indicated by black squares.

**Figure 12:**
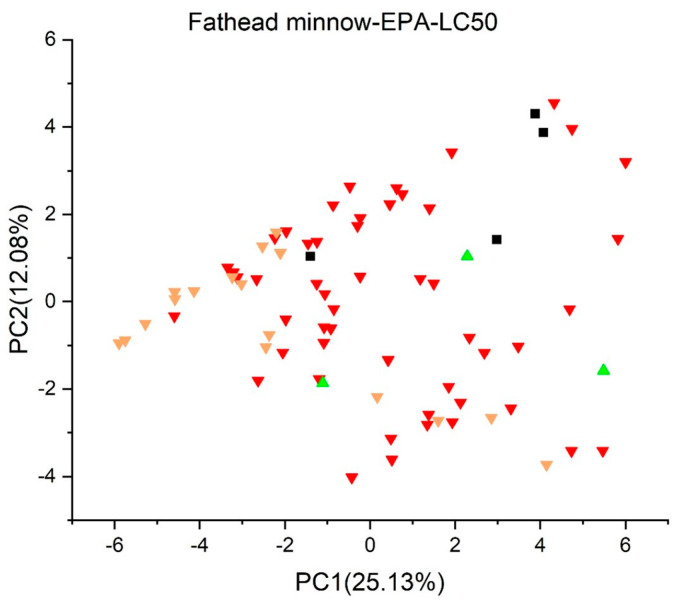
Score plot for Fathead Minnow LC50 96 h (EPA). Compounds are color−coded based on their LC50 values: toxicity below 1 mg/L is in red, between 1 mg/L and 10 mg/L in yellow, and over 10 mg/L in green. Compounds with disconnected structures are indicated by black squares.

**Figure 13:**
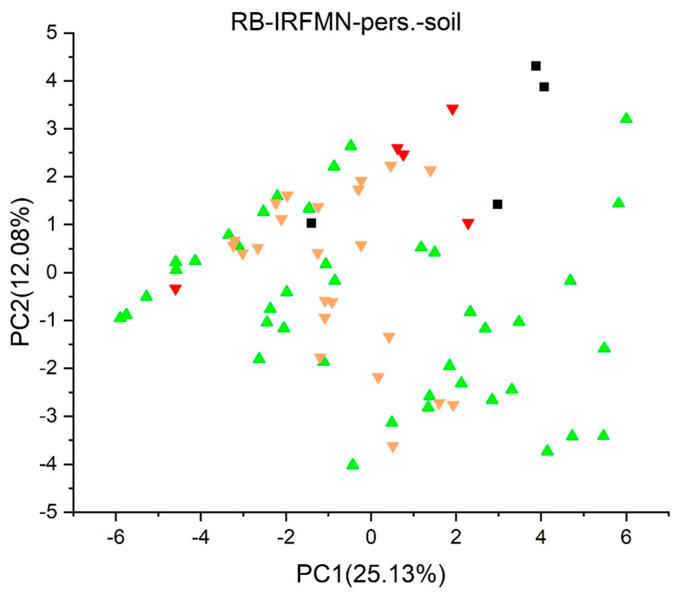
Score plot for Persistence (soil) quantitative model (IRFMN). Compounds are color−coded as follows: green non-persistent (nP), yellow persistent/non-persistent (P/nP), red very-persistent (vP). Compounds with disconnected structures are indicated by black squares.

**Figure 14:**
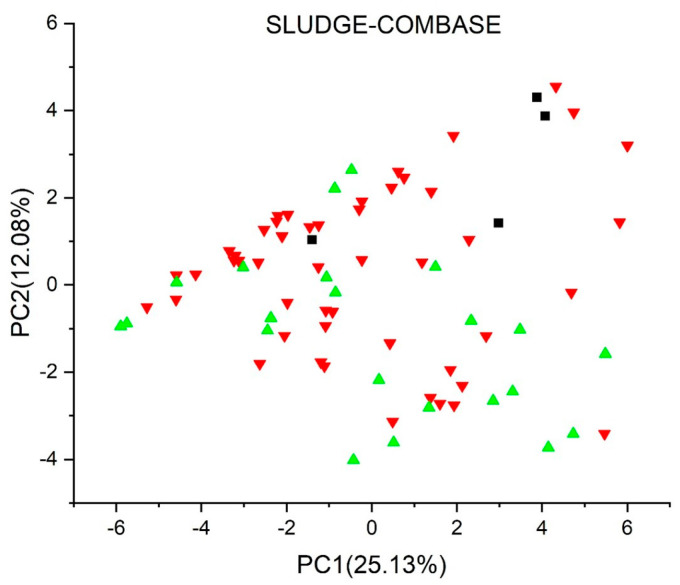
Score plot for Sludge Classification Toxicity model (ProtoQSAR/COMBASE). Compounds are color−coded based on their prediction as follows: toxic in red and non−toxic in green. Compounds with disconnected structures are indicated by black squares.

**Figure 15:**
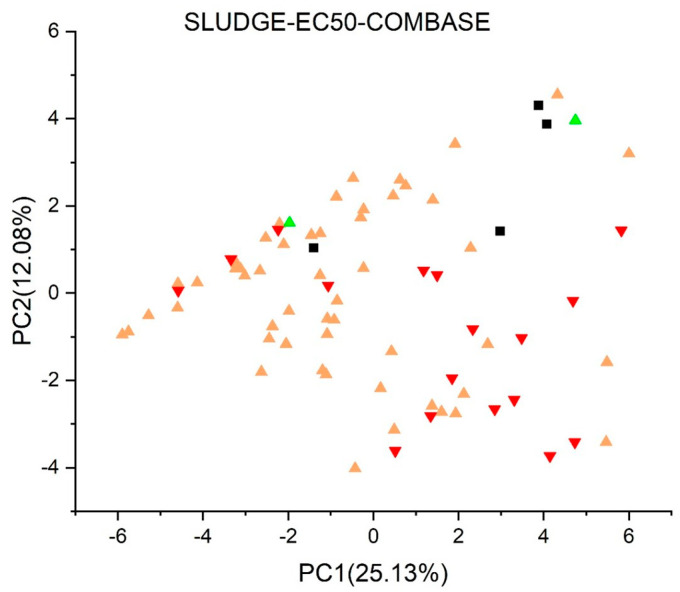
Score plot for Sludge (EC50) toxicity model (ProtoQSAR/COMBASE). Compounds are color−coded based on their EC50 values: below 10 mg/L in red, between 10 and 100 mg/L in yellow, and over 100 mg/L in green. Compounds with disconnected structures are indicated by black squares.

Figure 3, Figure 4, Figure 5, Figure 6, Figure 7, Figure 8, Figure 9, Figure 10, Figure 11, Figure 12, Figure 13, Figure 14 and Figure 15 show the score plots corresponding to the ecotoxicity models selected for the analysis, which present the objects (structures) in dependence on PC1 and PC2. It is evident that the compounds are evenly distributed over the maps, and no particular clusters can be seen. However, in certain models, compounds with similar activity levels (same color) tend to aggregate in specific regions of the maps. The four compounds with disconnected structures are represented by black squares. It is worth noting that these compounds were excluded from further analysis.

Figure 3, Figure 4, Figure 5 and Figure 6 illustrate the score plots of four models designed for predicting the bioconcentration factor (BCF). Compounds with a BCF value below 100 L/kg are categorized as of no concern and are denoted in green, those falling between 100 and 1000 L/kg in yellow, and those exceeding 1000 L/kg in red. In Figure 3, illustrating the *BCF model* (*Arnot-Gobas*), the line PC1 = 0 divides the map into two sections. The left segment, defined by the condition PC1 < 0, contains 37 compounds, out of which 8 (21.6%) are predicted to be of low concern, labeled green in the plot. Moving to Figure 4, the *BCF model* (*CAESAR*) predominantly predicts compounds with a BCF below 100 L/kg, and no compound exceeds 1000 L/kg. However, 16 compounds with a BCF > 100 L/kg (yellow color) are concentrated in the region where PC1 < 0.

When examining the results from the four Bioconcentration Factor (BCF) models, it becomes clear that a conclusive consensus report cannot be drawn. Among these models, only two predict bioconcentration factors exceeding 1000 L/kg for certain compounds. The lack of unanimity among the BCF models underscores the complexity and variability in predicting the bioaccumulation potential of the studied compounds. This divergence in predictions emphasizes the importance of cautious interpretation and further investigation to gain a comprehensive understanding of the potential environmental implications associated with these compounds.

In Figure 5, representing the *BCF model* (*kNN/Read Across*), 32 compounds are predicted to have a BCF between 100 and 1000 L/kg and no compounds with a BCF higher than 1000 L/kg. Meanwhile, in Figure 6, the *BCF model* (*Meylan*) predicts 33 compounds with BCF larger than 1000 L/kg, with 10 of them exceeding 10,000 L/kg.

For algae toxicity, three models were included in the analysis, and their score plots are depicted in Figure 7, Figure 8 and Figure 9. In Figure 7 and Figure 8, compounds with a toxic dose EC50 less than 1.0 mg/L are denoted in red, those falling between 1.0 and 10 mg/L in yellow, and those with a LC50 greater than 10 mg/L in green. Figure 9 showcases compounds with NOEC under 0.1 mg/L in red, those with a dose between 0.1 mg/L and 1.0 mg/L in yellow, and those with a dose over 1.0 mg/L in green.

Within the score plot of the *Algae* (*EC50*) *Toxicity Model* (*ProtoQSAR/Combase*) in Figure 7, there is an area PC2 < 2 mostly populated with non-toxic compounds, a pattern similarly supported by the *Algae Chronic* (*NOEC*) *Toxicity model* (*IRFMN*) plot in Figure 9. In the *Algae* (*EC50*) *Toxicity Model* (*IRFMN*), 35 compounds are colored red, 34 yellow, and three green. The score plot (Figure 8) shows that compounds are equally distributed over the map.

In Figure 10, *Daphnia Acute* (*EC50*) *toxicity model* (*IRFMN*), the compounds with EC50 under 0.1 mg/L are labeled in red, those between 0.1 and 1.0 mg/L in yellow, and those above 1.0 mg/L (of low concern) are green. Most compounds in the region PC1 < 0 are deemed of low concern.

Regarding the *Fish Acute* (*LC50*) *Toxicity model* (*IRFMN*) in Figure 11, most of the compounds have a toxicity below 10 mg/L, except for BPF (4,4′-methylendiphenol), which displays a toxicity of 10.7 mg/L. The *Fathead Minnow LC50 96 h* (*EPA*) (Figure 12) shows similar traits; however, three compounds have LC50s over 10 mg/L; bisphenol A bis(2-hydroxyethyl)ether (BPA 2EO), tetrakis(dimethylaminomethyl)bisphenol A, and 5′-O-(p,p′-dimethoxytrityl)thymidine (DMT-T).

Upon careful examination of the results obtained from both fish toxicity models, a concluding consensus report cannot be formulated. The predictions from these models exhibit a lack of uniformity, with divergent outcomes for various compounds. Further investigation and consideration of additional factors may be essential for a more comprehensive understanding of the potential ecological implications and fish toxicity associated with the compounds under study.

Figure 13 shows the *Persistence* (*soil*) *quantitative model* (*IRFMN*), with five compounds predicted as persistent to very persistent, 23 as non-persistent to persistent, and the majority (42 compounds) as non-persistent. Two compounds were not predicted by the model. In the *Sludge* (*EC50*) *toxicity* (*ProtoQSAR/COMBASE*) (Figure 15), 17 compounds have predicted EC50 values below 10 mg/L, 43 fall between 10 and 100 mg/L, and two have EC50 values over 100 mg/L, each labeled in red, yellow, and green, respectively. Regarding the *Sludge Classification Toxicity model* (*ProtoQSAR/COMBASE*) presented in Figure 14. The compounds predicted to be toxic or non-toxic are labeled red or green, respectively.

**Figure 16:**
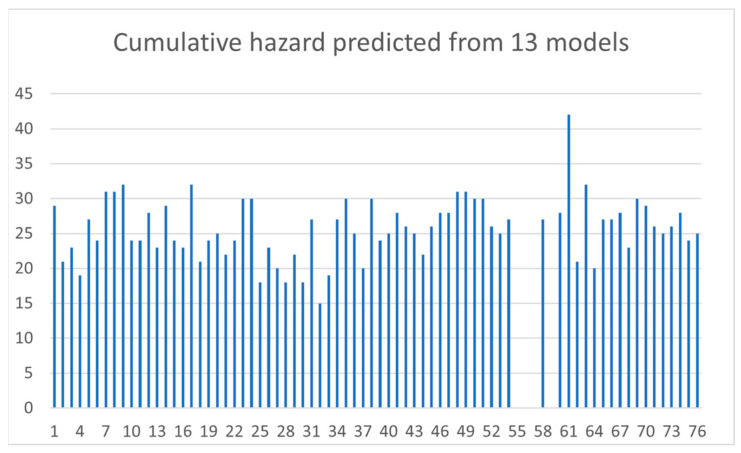
Cumulative hazard predicted by the in silico ecotoxicological models.

Finally, we evaluated the obtained ecotoxicological profiles with a cumulative factor. Each prediction was assigned a numerical value (1, 2, or 3) corresponding to its toxicity level, with 3 indicating the most toxic compounds—represented by the red labels in Figure 3, Figure 4, Figure 5, Figure 6, Figure 7, Figure 8, Figure 9, Figure 10, Figure 11, Figure 12, Figure 13, Figure 14 and Figure 15, 2 indicating moderate toxicity, and 1 signifying non-toxic substances. The sums over all 13 models for individual compounds are displayed in Figure 16. Fifteen compounds resulted in a sum of over 30. The highest value lying at score 42 is attributed to the compound p,p′-(2-pyridylmethylene)bisphenol (DDPM). As emphasized by Ballabio et al. [59], consensus prediction, derived from diverse models constructed on distinct datasets and employing different methodologies, may provide enhanced reliability compared to individual models.

It is crucial to highlight that the evaluation conducted focused exclusively on the predicted ecotoxicological profile of DDPM. In a broader context, DDPM, along with other BPA alternatives, is currently under consideration by the European Chemicals Agency (ECHA) in the assessment of regulatory needs [60]. According to this document, currently, the available information is insufficient to assess the necessity for regulatory risk management. Key areas such as reproductive toxicity, endocrine-disrupting (ED) properties, skin sensitization, and persistent, bioaccumulative, and toxic (PBT)/very persistent and very bioaccumulative (vPvB) characteristics for 65 bisphenols lack adequate data for conclusive judgments.

ECHA acknowledges that ongoing data generation efforts on other bisphenols may inform future regulatory actions. However, potential challenges, including structural variations and variations in reactivity due to different functional groups, could impact the feasibility of employing read-across approaches. It is noteworthy that DDPM is registered as an intermediate under Art. 17/18, limiting further data generation under Dossier Evaluation. Additionally, substance evaluation for DDPM faces challenges, given the general lack of close structural relations to other bisphenols that could justify concerns, coupled with either low tonnages or low exposure potential [60].

## 4. Conclusions

We present the ecotoxicological study of bisphenol A and its potential alternatives. The selection of models featuring bisphenol derivatives in their training data was performed on the VEGA HUB platform. Regardless of the chosen models, a principal component analysis (PCA) of the data was conducted, considering exclusively the molecular structures of the compounds, as these molecules were represented by numerous structural descriptors. The analysis showed that no particular group of descriptors carries the essential part of the variance within the dataset.

The analysis of score plots reveals that no particular clusters are formed. Nonetheless, certain score plot areas populated with predominately toxic or non-toxic compounds can be identified. The position and neighbor compounds in the score plot can provide additional support for the predictions. Additionally, we assessed the predictions for each compound and model, assigning a value from 1 to 3 based on the predicted toxicity level. The cumulative score across models serves as a comprehensive evaluation of the ecotoxicity of each individual compound.

Notably, the highest score of 42 was achieved by p,p′-(2-pyridylmethylene)bisphenol (DDPM). Additionally, 14 other compounds reached a score over 30: allyl bisphenol A (DAB), tetramethylbisphenol A (TMBPA), BAPP, bisphenol A bisallyl ether, BPG, BPPH, 4,4′-methylenebis(2,6- dimethylphenyl cyanate), BPAP, BPM, BPP, BP-TMC, BPZ, DMT-Cl, and BisP-IOTD. These compounds were predicted to exhibit higher toxicity at the selected endpoints, potentially indicating a greater environmental concern.

While each of the models studied can be used to predict the ecotoxicity of bisphenol derivatives, it’s important to note that they are not exclusively trained on this specific class of compounds, so their predictions should be carefully evaluated. This underscores the clear need for more experimental data, which can be used to develop models that are more specific to BPA alternatives.

## Data Availability

Data is contained within the article or Supplementary Materials.

## Supplementary Materials

The following supporting information can be downloaded at: https://www.mdpi.com/article/10.3390/jox13040046/s1, Table S1: Dataset of bisphenol alternatives utilized in the study.

## References

[1] Geens T., Aerts D., Berthot C., Bourguignon J.-P., Goeyens L., Lecomte P., Maghuin-Rogister G., Pironnet A.-M., Pussemier L., Scippo M.-L. (2012). A review of dietary and non-dietary exposure to bisphenol-A. Food Chem. Toxicol..

[2] Lassen C., Brandt U.K. (2011). Migration of Bisphenol A from Cash Register Receipts and Baby Dummies.

[3] Vandenberg L.N., Hauser R., Marcus M., Olea N., Welshons W.V. (2007). Human exposure to bisphenol A (BPA). Reprod. Toxicol..

[4] Cao X.-L., Perez-Locas C., Dufresne G., Clement G., Popovic S., Beraldin F., Dabeka R.W., Feeley M. (2011). Concentrations of bisphenol A in the composite food samples from the 2008 Canadian total diet study in Quebec City and dietary intake estimates. Food Addit. Contam. Part A.

[5] Cunha S., Fernandes J. (2013). Assessment of bisphenol A and bisphenol B in canned vegetables and fruits by gas chromatography–mass spectrometry after QuEChERS and dispersive liquid–liquid microextraction. Food Control.

[6] Noonan G.O., Ackerman L.K., Begley T.H. (2011). Concentration of Bisphenol A in Highly Consumed Canned Foods on the U.S. Market. J. Agric. Food Chem..

[7] Gong X., Xie H., Li X., Wu J., Lin Y. (2017). Bisphenol A induced apoptosis and transcriptome differences of spermatogonial stem cells in vitro. Acta Biochim. Biophys. Sin..

[8] Harnett K.G., Moore L.G., Chin A., Cohen I.C., Lautrup R.R., Schuh S.M. (2021). Teratogenicity and toxicity of the new BPA alternative TMBPF, and BPA, BPS, and BPAF in chick embryonic development. Curr. Res. Toxicol..

[9] Jagne J., White D., Jefferson F. (2016). Endocrine-Disrupting Chemicals: Adverse Effects of Bisphenol A and Parabens to Women’s Health. Water Air Soil Pollut..

[10] Pouzaud F., Thierry-Mieg M., Burga K., Vérines-Jouin L., Fiore K., Beausoleil C., Michel C., Rousselle C., Pasquier E. (2018). Concerns related to ED-mediated effects of Bisphenol A and their regulatory consideration. Mol. Cell. Endocrinol..

[11] Rubin B.S. (2011). Bisphenol A: An endocrine disruptor with widespread exposure and multiple effects. J. Steroid Biochem. Mol. Biol..

[12] Chen D., Kannan K., Tan H., Zheng Z., Feng Y.-L., Wu Y., Widelka M. (2016). Bisphenol Analogues Other Than BPA: Environmental Occurrence, Human Exposure, and Toxicity—A Review. Environ. Sci. Technol..

[13] Eladak S., Grisin T., Moison D., Guerquin M.-J., N’Tumba-Byn T., Pozzi-Gaudin S., Benachi A., Livera G., Rouiller-Fabre V., Habert R. (2015). A new chapter in the bisphenol A story: Bisphenol S and bisphenol F are not safe alternatives to this compound. Fertil. Steril..

[14] Rochester J.R., Bolden A.L. (2015). Bisphenol S and F: A Systematic Review and Comparison of the Hormonal Activity of Bisphenol A Substitutes. Environ. Health Perspect..

[15] Kojima H., Takeuchi S., Sanoh S., Okuda K., Kitamura S., Uramaru N., Sugihara K., Yoshinari K. (2019). Profiling of bisphenol A and eight of its analogues on transcriptional activity via human nuclear receptors. Toxicology.

[16] Liu B., Lehmler H.-J., Sun Y., Xu G., Sun Q., Snetselaar L.G., Wallace R.B., Bao W. (2019). Association of Bisphenol A and Its Substitutes, Bisphenol F and Bisphenol S, with Obesity in United States Children and Adolescents. Diabetes Metab. J..

[17] Marx-Stoelting P., Rivière G., Luijten M., Aiello-Holden K., Bandow N., Baken K., Cañas A., Castano A., Denys S., Fillol C. (2023). A walk in the PARC: Developing and implementing 21st century chemical risk assessment in Europe. Arch. Toxicol..

[18] Nowak K., Jakopin Ž. (2023). In silico profiling of endocrine-disrupting potential of bisphenol analogues and their halogenated transformation products. Food Chem. Toxicol..

[19] EC (2016). Commission Regulation (EU) 2016/2235 of 12 December 2016 amending Annex XVII to Regulation (EC) No 1907/2006 of the European Parliament and of the Council concerning the Registration, Evaluation, Authorisation and Restriction of Chemicals (REACH). https://eur-lex.europa.eu/legal-content/EN/TXT/PDF/?uri=CELEX:32016R2235&from=EN.

[20] EFSA (2021). Bisphenol A: EFSA Draft Opinion Proposes Lowering the Tolerable Daily Intake.

[21] Ou-Yang S., Lu J., Kong X., Liang Z., Luo C., Jiang H. (2012). Computational drug discovery. Acta Pharmacol. Sin..

[22] Xiang M., Cao Y., Fan W., Chen L., Mo Y. (2012). Computer-Aided Drug Design: Lead Discovery and Optimization. Comb. Chem. High Throughput Screen..

[23] EC (2006). Regulation (EC) No 1907/2006 of the European Parliament and of the Council of 18 December 2006 Concerning the Registration, Evaluation, Authorisation and Restriction of Chemicals (REACH). https://eur-lex.europa.eu/legal-content/EN/TXT/PDF/?uri=CELEX:02006R1907-20161011&from=EN.

[24] TSCA (2021). Toxic Substances Control Act (TSCA). https://www.congress.gov/bill/114th-congress/senate-bill/697/all-info.

[25] FDA (2016). Use of International Standard ISO 10993-1. “Biological Evaluation of Medical Devices—Part 1: Evaluation and Testing within a Risk Management Process”. Guidance for Industry and Food and Drug Administration Staff. https://www.fda.gov/regulatory-information/search-fda-guidance-documents/use-international-standard-iso-10993-1-biological-evaluation-medical-devices-part-1-evaluation-and.

[26] FDA (2016). Premarket Tobacco Product Applications for Electronic Nicotine Delivery Systems. Guidance for Industry. https://www.fda.gov/media/97652/download.

[27] ECHA (2008). Guidance on the Application of the CLP Criteria. Guidance to Regulation (EC) No 1272/2008 on Classification, Labelling and Packaging (CLP) of Substances and Mixtures. https://echa.europa.eu/documents/10162/23036412/clp_en.pdf/58b5dc6d-ac2a-4910-9702-e9e1f5051cc5.

[28] Freidig A., Dekkers S., Verwei M., Zvinavashe E., Bessems J., van de Sandt J. (2007). Development of a QSAR for worst case estimates of acute toxicity of chemically reactive compounds. Toxicol. Lett..

[29] EC (2009). Guidance Document on the Assessment of the Equivalence of Technical Materials of Substances Regulated under Regulation (EC) No 1107/2009 SANCO/10597/2003. https://food.ec.europa.eu/system/files/2016-10/pesticides_guidance_equivalence-chem-substances_en.pdf.

[30] SCCS (2016). Memorandum on the Use of in Silico Methods for Assessment of Chemical Hazard. SCCS/1578/16. Scientific Committee on Consumer Safety.

[31] Mauri A., Roy K. (2020). alvaDesc: A Tool to Calculate and Analyze Molecular Descriptors and Fingerprints. Ecotoxicological QSARs.

[32] Mauri A., Bertola M. (2022). Alvascience: A New Software Suite for the QSAR Workflow Applied to the Blood–Brain Barrier Permeability. Int. J. Mol. Sci..

[33] Benfenati E., Manganaro A., Gini G. (2013). VEGA-QSAR: AI inside a platform for predictive toxicology. PAI@ AI* IA.

[34] Benfenati E., Colombo E. (2022). Fish Acute (LC50) Toxicity model (IRFMN) (version 1.0.1). QSAR Model Reporting Format (QMRF).

[35] Toma C., Cappelli C.I., Manganaro A., Lombardo A., Arning J., Benfenati E. (2021). New Models to Predict the Acute and Chronic Toxicities of Representative Species of the Main Trophic Levels of Aquatic Environments. Molecules.

[36] (2016). Results of aquatic toxicity tests of chemicals conducted by Ministry of the Environment in Japan (March 2016). Series Results of Aquatic Toxicity Tests of Chemicals Conducted by Ministry of the Environment in Japan (March 2016).

[37] Benfenati E., Colombo E. Fathead Minnow LC50 96h (EPA) (version 1.0.8). In *QSAR Model Reporting Format (QMRF)*; Vegahub: 2022. https://www.vegahub.eu/vegahub-dwn/qmrf/QMRF_FATHEAD_LC50_EPA.pdf.

[38] Martin T.M., Young D.M. (2001). Prediction of the Acute Toxicity (96-h LC_50_) of Organic Compounds to the Fathead Minnow (*Pimephales promelas*) Using a Group Contribution Method. Chem. Res. Toxicol..

[39] Benfenati E., Cappelli C.I., Toma C. (2019). Daphnia Acute (EC50) toxicity model (IRFMN) v1.0.1. QSAR Model Reporting Format (QMRF).

[40] Benfenati E., Gamba A. (2021). Algae Acute (EC50) Toxicity model (IRFMN)—V1.0.1. QSAR Model Reporting Format (QMRF).

[41] Blázquez M., Andreu-Sánchez O., Ballesteros A., Fernández-Cruz M.L., Fito C., Gómez-Ganau S., Gozalbes R., Hernández-Moreno D., Julián-Ortiz D., Vicente J. (2021). Computational Tools for the Assessment and Substitution of Biocidal Active Substances of Ecotoxicological Concern. Chemometrics and Cheminformatics in Aquatic Toxicology.

[42] Gómez-Ganau S., Gozalbes R. (2019). Algae (EC50) Toxicity Model (ProtoQSAR/Combase) v.1.0.1. QSAR Model Reporting Format (QMRF).

[43] Benfenati E., Manganaro A. (2022). Algae Chronic (NOEC) Toxicity model (IRFMN)—V.1.0.1. QSAR Model Reporting Format (QMRF).

[44] Gómez-Ganau S. (2022). Sludge Classification Toxicity model (ProtoQSAR/COMBASE) (Version 1.0.1). QSAR Model Reporting Format (QMRF).

[45] Gómez-Ganau S., Marzo M., Gozalbes R., Benfenati E., Roy K. (2020). Computational Approaches to Evaluate Ecotoxicity of Biocides: Cases from the Project COMBASE. Ecotoxicological QSARs.

[46] Gómez-Ganau S. (2022). Sludge (EC50) toxicity (ProtoQSAR/COMBASE) (Version 1.0.1). QSAR Model Reporting Format (QMRF).

[47] Lombardo A., Roncaglioni A., Boriani E., Milan C., Benfenati E. (2010). Assessment and validation of the CAESAR predictive model for bioconcentration factor (BCF) in fish. Chem. Cent. J..

[48] Zhao C., Boriani E., Chana A., Roncaglioni A., Benfenati E. (2022). Model to predict bioconcentration factors (BCF) v 2.1.15 (CAESAR). QSAR Model Reporting Format (QMRF).

[49] Zhao C., Boriani E., Chana A., Roncaglioni A., Benfenati E. (2008). A new hybrid system of QSAR models for predicting bioconcentration factors (BCF). Chemosphere.

[50] Dimitrov S., Dimitrova N., Parkerton T., Comber M., Bonnell M., Mekenyan O. (2005). Base-line model for identifying the bioaccumulation potential of chemicals. SAR QSAR Environ. Res..

[51] Arnot J.A., Gobas F.A.P.C. (2003). A Generic QSAR for Assessing the Bioaccumulation Potential of Organic Chemicals in Aquatic Food Webs. QSAR Comb. Sci..

[52] Benfenati E., Manganaro A. (2020). BCF model (Arnot-Gobas)—V.1.0.1. QSAR Model Reporting Format (QMRF).

[53] Meylan W.M., Benfenati E. (2019). BCF model (Meylan) v 1.0.4. QSAR Model Reporting Format (QMRF).

[54] Meylan W.M., Howard P.H., Boethling R.S., Aronson D., Printup H., Gouchie S. (1999). Improved method for estimating bioconcentration/bioaccumulation factor from octanol/water partition coefficient. Environ. Toxicol. Chem..

[55] Manganaro A., Benfenati E. (2020). A BCF model (kNN/Read-Across) v 1.1.1. QSAR Model Reporting Format (QMRF).

[56] Manganaro A., Pizzo F., Lombardo A., Pogliaghi A., Benfenati E. (2016). Predicting persistence in the sediment compartment with a new automatic software based on the k-Nearest Neighbor (k-NN) algorithm. Chemosphere.

[57] Lombardo A., Benfenati E., Manganaro A. (2022). Persistence (soil) quantitative model (IRFMN)—V.1.0.1. QSAR Model Reporting Format (QMRF).

[58] Gouin T., Cousins I., Mackay D. (2004). Comparison of two methods for obtaining degradation half-lives. Chemosphere.

[59] Ballabio D., Biganzoli F., Todeschini R., Consonni V. (2017). Qualitative consensus of QSAR ready biodegradability predictions. Toxicol. Environ. Chem..

[60] ECHA (2021). Bisphenols. Assessment of Regulatory Needs.

