# Supplementary material for: Ecotoxicological Evaluation of Bisphenol A and Alternatives: A Comprehensive In Silico Modelling Approach
Source: J Xenobiot. 2023 Nov 23;13(4):719–39. doi: 10.3390/jox13040046 (PMC10744758; doi:10.3390/jox13040046)
Supplement: Supplementary file 1 [file jox-13-00046-s001.zip › jox-2698857-supplementary.pdf]

Journal of Xenobiotics

Supplementary Materials for

Ecotoxicological Evaluation of Bisphenol A and alternatives: A Comprehensive in silico Modelling Approach

Liadys Mora Lagares[1,] *, and Marjan Vračko[1]

[1] Theory Department, Laboratory for Cheminformatics, National Institute of Chemistry, 1000 Ljubljana, Slovenia; marjan.vracko@ki.si

* Correspondence: liadys.moralagares@ki.si (L.M.L.) Tel.: +386-1-4760-439

Table S1. Dataset of bisphenol alternatives utilized in the study.

ID	EC/List No	CAS	Chemical Name	Common name
1	201-240-0	79-97-0	4,4′-isopropylidenedi-o-cresol	3,3'-Dimethylbisphenol A; Dicresylolpropane, BPC
2	201-245-8	80-05-7	4,4′-isopropylidenediphenol	BPA, bisphenol A
3	204-137-9	116-37-0	1,1′-isopropylidenebis(p-phenyleneoxy)dipropan-2-ol	Bisphenol A bis(2-hydroxypropyl) ether; BPA 2 PO
4	212-985-6	901-44-0	2,2′-isopropylidenebis(p-phenyleneoxy)diethanol	Bisphenol A bis(2-hydroxyethyl)ether; BPA 2 EO
5	214-590-4	1156-51-0	4,4′-isopropylidenediphenyl dicyanate	Bisphenol A cyanate ester; BADCy
6	216-823-5	1675-54-3	2,2′-[(1-methylethylidene)bis(4,1-phenyleneoxymethylene)]bisoxirane	BISPHENOL A DIGLYCIDYL ETHER; BADGE
7	217-121-1	1745-89-7	4,4′-isopropylidenebis[2- allylphenol]	Allyl bisphenol A; DAB
8	227-033-5	5613-46-7	4,4′-isopropylidenedi-2,6-xylol	Tetramethylbisphenol A; TMBPA
9	235-985-8	13080-86-9	4,4′-[isopropylidenebis(4,1- phenyleneoxy)]dianiline	BAPP
10	242-895-2	19224-29-4	2,2′-[(1-methylethylidene)bis(4,1-phenyleneoxy)]bisethyl diacetate	SCHEMBL11889494, EINECS 242-895-2
11	248-607-1	27689-12-9	(1-methylethylidene)bis(4,1- phenyleneoxy-3,1-propanediyl) bismethacrylate	2,2-bis-(4-(3-methacryloxypropoxy)phenyl)propane; BIS-PMA
12	253-781-7	38103-06-9	4,4′-[(isopropylidene)bis(p- phenyleneoxy)]diphthalic dianhydride	4,4'-Bisphenol A dianhydride; BPA-DA
13	425-220-8	5945-33-5	(1-methylethylidene)di-4,1- phenylene tetraphenyl diphosphate	Bisphenol A bis(diphenyl phosphate); BPA-DP
14	432-380-2	147504-92-5	4,6-bis[2-(4-hydroxyphenyl)isopropylidene]resorcinol	HBPX-1
15	434-000-0	n/a	3-{2-[2,4-dihydroxy-5-(2-phenylpropan-2-yl)phenyl]propan-2-yl}phenyl 6-(-lambda5-diazynylidene)-5-methylidene-4a,5,6,8a-tetrahydronaphthalene-1-sulfonate	PURAM

16	216-367-7	1565-94-2	(1-methylethylidene)bis[4,1- phenyleneoxy(2-hydroxy-3,1- propanediyl)] bismethacrylate	Silux; Bisphenol A glycidylmethacrylate
17	223-123-3	3739-67-1	4,4′-isopropylidenebis[(allyloxy)benzene]	Bisphenol A bisallyl ether; 2,2-Bis(4-allyloxyphenyl)propane
18	225-144-3	4687-94-9	(1-methylethylidene)bis[4,1- phenyleneoxy(2-hydroxy-3,1- propanediyl)] diacrylate	Bisphenol a diglycidyl ether diacrylate
19	246-263-7	24448-20-2	(1-methylethylidene)bis(4,1- phenyleneoxy-2,1-ethanediyl) bismethacrylate	Bisphenol A bis(2-hydroxyethyl ether) dimethacrylate
20	605-281-9	16224-36-5	4,4-(1-Methylethyliden)-bis- [2,6-bis- (dimethylaminomethyl)-phenol]	Tetrakis(dimethylaminomethyl)bisphenol A
21	605-913-3	181028-79-5	[4-[2-(4-phosphonooxyphenyl)propan-2-yl]phenyl] dihydrogen phosphate	Bisphenol A diphosphate
22	613-584-2	64401-02-1	2,2-Bis[4-(2-Acryloxyethoxy)phenyl]propane	Ethoxylated (2) bisphenol A diacrylate
23	678-196-8	127-54-8	2,2-Bis(4-hydroxy-3-isopropylphenyl)propane	Bisphenol G; BPG
24	678-197-3	24038-68-4	2,2-Bis(2-hydroxy-5-biphenylyl)propane	Bisphenol PH; BPPH
25	201-250-5	80-09-1	4,4'-sulphonyldiphenol	BPS
26	235-986-3	13080-89-2	4,4'-[sulphonylbis(4,1-phenyleneoxy)]dianiline	n/a
27	263-920-3	63134-33-8	p-[[p- benzyloxyphenyl]s ulphonyl]phenol	BPS-MPE
28	405-520-5	95235-30-6	4-(4-isopropoxyphenyls ulfonyl)phenol	D8 (D88); D8(HPS); BPS-monoP
29	411-570-9	41481-66-7	2,2'-diallyl-4,4'- sulfonyldiphenol	TG-SB, TG-SH, TG- SH(H)
30	479-880-7	97042-18-7	4-(4-Allyloxy-benzenesulfonyl)-phenol	BPS-MAE
31	204-279-1	118-82-1	2,2',6,6'-tetra-tert-butyl- 4,4'-methylenediphenol	TBMD; 4,4'-Methylenebis(2,6-DI-tert-butylphenol)
32	210-658-2	620-92-8	4,4'-methylenediphenol	BPF
33	218-257-4	2095-03-6	2,2'-[methylenebis(p-phenyleneoxymethylene)]bisoxirane	Bisphenol F diglycidyl ether; Bis(4-glycidyloxyphenyl)methane
34	226-378-9	5384-21-4	4,4'-methylenedi-2,6-xylenol	Tetramethyl Bisphenol F
35	405-790-4	101657-77-6	4,4'-methylenebis(2,6-dimethylphenylcyanate)	n/a

36	439-910-1	93705-66-9	2-[[4-[[3,5-dimethyl-4-(oxiran-2-ylmethoxy)phenyl]methyl]-2,6-dimethylphenoxy]methyl]oxirane	YSLV-80XY
37	908-912-9	1333-16-0	2-[(2-hydroxyphenyl)methyl]phenol	2,2'-Bisphenol F
38	433-130-5	1571-75-1	4-[1-(4-hydroxyphenyl)-1-phenylethyl]phenol	Bisphenol AP; BPAP
39	811-683-7	1799707-26-8	4,4'-(1-phenylethane-1,1-diyl)bis(heptyloxybenzene)	n/a
40	201-025-1	77-40-7	4-[2-(4-hydroxyphenyl)butan-2-yl]phenol	Bisphenol B; BPB
41	679-999-6	1844-01-5	4,4'-Dihydroxytetraphenylmethane	BPBP
42	238-940-0	14868-03-2	4-[2,2-dichloro-1-(4-hydroxyphenyl)ethenyl]phenol	Bisphenol C; BPC2 (BPCl2)
43*	945-909-1	69415-01-6	bis(2-{[4-(2,2-dichloro-1-{4-[(oxiran-2-yl)methoxy]phenyl}ethenyl)phenoxy]methyl}oxirane)	1,1-Bis(p-hydroxyphenyl)-2,2-dichloroethylene diglycidyl ether
44	627-637-2	2081-08-5	4-[1-(4-hydroxyphenyl)ethyl]phenol	Bisphenol E; BPE
45	405-740-1	47073-92-7	4,4'-ethylidenediphenyl dicyanate	1,1-Bis(4-cyanatophenyl)ethane
46	404-470-1	n/a	2-{[4-(9-{4-[(oxiran-2-yl)methoxy]phenyl}-9H-fluoren-9-yl)phenoxy]methyl}oxirane	EPIKOTE 1079
47	406-950-6	3236-71-3	9,9-bis(4- hydroxyphenyl)fluorene	BPFL; Fluorene-9-bisphenol
48	428-970-4	13595-25-0	4,4'-(1,3-phenylene-bis(1- methylethylidene))bis-pheno	Bisphenol M; BPM
49	606-820-0	2167-51-3	4,4'-(1,4-Phenylenediisopropylidene)bisphenol	Bisphenol P; BPP
50	404-140-7	129188-99-4	1,1-Bis(4-hydroxyphenyl)-3,3,5-trimethylcyclohexane	BISPHENOL TMC; BP-TMC
51	212-677-1	843-55-0	4,4'-cyclohexylidenebisphenol	Bisphenol Z; BPZ
52	219-110-7	2362-14-3	Phenol, 4,4'-cyclohexylidenebis[2-methyl-	4,4'-cyclohexylidenedi-o-cresol
53	810-464-3	13446-84-9	2,2'-[cyclohexane-1,1- diylbis(4,1-phenyleneoxymethylene)]dioxirane	1,1-Bis[4-(glycidyloxy)phenyl]cyclohexane
54	216-036-7	1478-61-1	4,4'-[2,2,2-trifluoro-1-(trifluoromethyl)ethylidene]diphenol	Bisphenol AF; BPAF
55*	278-305-5	75768-65-9	benzyltriphenylphosphonium,salt with 4,4'-[2,2,2-trifluoro-1-(trifluoromethyl)ethylidene]bis[phenol] (1:1)	BPAF-salt
56	425-060-9	n/a	disodium 4-[1,1,1,3,3,3-hexafluoro-2-(4-oxidophenyl)propan-2-yl]benzen-1-olate	T-6627

57*	468-740-0	n/a	Tributyl-2-methoxypropylphosphonium salt with 4,4'-[2,2,2- trifluoro-1-(trifluoromethyl)ethylidene]bis[phenol]	BPAF-salt
58	469-080-6	1478-61-1	4-[1,1,1,3,3,3-hexafluoro-2-(4-hydroxyphenyl)propan-2-yl]phenol	BPAF-salt
59*	479-100-5	577705-90-9	benzyl(diethylamino)diphenylphosphonium 4-[1,1,1,3,3,3-hexafluoro- 2-(4-hydroxyphenyl)propan- 2-yl]phenolate	BPAF-salt
60	201-618-5	85-60-9	6,6'-di-tert-butyl-4,4'-butylidenedi-m-cresol	Santowhite
61	210-039-7	603-41-8	p,p'-(2-pyridylmethylene)bisphenol	DDPM
62	217-420-7	1843-03-4	4,4',4''-(1-methylpropanyl-3-ylidene)tris[6-tert-butyl-m-cresol]	Topanol CA
63	255-002-6	40615-36-9	1,1'-(chlorophenylmethylene)bis[4-methoxybenzene]	DMT-Cl
64	255-003-1	40615-39-2	5'-O-(p,p'-dimethoxytrityl)thymidine	DMT-T
65	401-720-1	n/a	2,2-bis(4'-hydroxyphenyl)-4-methylpentane	n/a
66	405-800-7	27955-94-8	4,4',4''-(ethan-1,1,1-triyl)triphenol	n/a
67	433-980-7	n/a	4-[1,3,5-tris(4-hydroxyphenyl)pentan-3-yl]phenol	CHEMICAL CODE NO 1592
68	610-104-3	43100-47-6	3-phenyl-5-(1,1,1-trifluoro-2-{6-hydroxy-5-phenyl-[1,1'-biphenyl]-3-yl}propan-2-yl)-[1,1'-biphenyl]-2-ol	n/a
69	680-046-1	74462-02-5	4,4'-(2-ethylhexane-1,1-diyl)diphenol	BisP-IOTD
70	201-236-9	79-94-7	2,6-dibromo-4-[2-(3,5-dibromo-4-hydroxyphenyl)propan-2-yl]phenol	Tetrabromobisphenol A; TBBPA
71	221-346-0	3072-84-2	2-[[2,6-dibromo-4-[2-[3,5-dibromo-4-(oxiran-2-ylmethoxy)phenyl]propan-2-yl]phenoxy]methyl]oxirane	Tetrabromobisphenol A diglycidyl ether; TBBPA-bGE
72	244-617-5	21850-44-2	1,3-dibromo-5-[2-[3,5-dibromo-4-(2,3-dibromopropoxy)phenyl]propan-2-yl]-2-(2,3-dibromopropoxy)benzene	Tetrabromobisphenol A bis(dibromopropyl ether); TBBPA-bDiBPrE

73	246-850-8	25327-89-3	1,3-dibromo-5-[2-(3,5-dibromo-4-prop-2-enoxyphenyl)propan-2-yl]-2-prop-2-enoxybenzene	Tetrabromobisphenol A diallyl ether; TBBPA-bAE
74	253-693-9	37853-61-5	1,3-dibromo-5-[2-(3,5-dibromo-4-methoxyphenyl)propan-2-yl]-2-methoxybenzene	Tetrabromobisphenol A dimethyl ether; TBBPA-bME
75	306-832-3	97416-84-7	1,1'-(Isopropylidene)bis(3,5-dibromo-4-(2,3-dibromo-2-methylpropoxy)benzene)	n/a
76	436-220-2	n/a	2,2-bis(3,5-dibromo-4-(3-acryloyloxy-2-hydroxypropoxy)phenyl)propane	n/a

* Compounds with disconnected structure.
